# Water-soluble polymer–drug conjugates for combination chemotherapy against visceral leishmaniasis

**DOI:** 10.1016/j.bmc.2010.02.043

**Published:** 2010-04-01

**Authors:** Salvatore Nicoletti, Karin Seifert, Ian H. Gilbert

**Affiliations:** a Division of Biological Chemistry and Drug Discovery, College of Life Sciences, University of Dundee, Sir James Black Centre, Dundee DD1 5EH, UK; b London School of Hygiene and Tropical Medicine, Keppel Street, London WC1E 7HT, UK

**Keywords:** Leishmaniasis, Polymer–drug conjugates, Combination therapy, Amphotericin B, Alendronate

## Abstract

There is a need for new safe, effective and short-course treatments for leishmaniasis; one strategy is to use combination chemotherapy. Polymer–drug conjugates have shown promise for the delivery of anti-leishmanial agents such as amphotericin B. In this paper, we report on the preparation and biological evaluation of polymer–drug conjugates of *N*-(2-hydroxypropyl)methacrylamide (HPMA), amphotericin B and alendronic acid. The combinatorial polymer–drug conjugates were effective anti-leishmanial agents in vitro and in vivo, but offered no advantage over the single poly(HPMA)–amphotericin B conjugates.

## Graphical abstract

**Figure:**
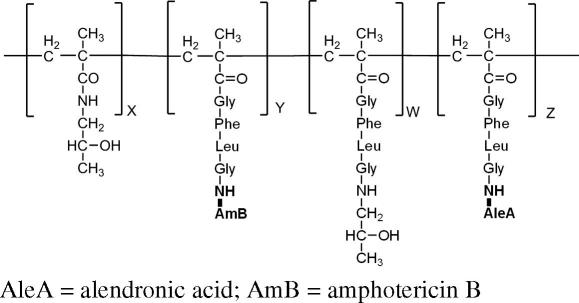

## 1 Introduction

Parasites of the genus *Leishmania* give rise to a number of different clinical manifestations. The most severe is visceral leishmaniasis (VL), which is almost always fatal unless treated. Estimated numbers of new VL cases are 500,000 per annum, with approximately 50,000 deaths each year, although VL is often not recognised or reported.^1^ Other forms of the disease, cutaneous leishmaniasis (CL), mucocutaneous leishmaniasis (MCL) and post-kala-azar dermal leishmaniasis (PKDL) are also major problems in many parts of the world.

Drugs remain the most important tool for both treatment and control of disease. Current drugs against VL include pentavalent antimonials (sodium stibogluconate (Pentostam®) and meglumine antimoniate), the polyene antibiotic amphotericin B (amphotericin B deoxycholate, Fungizone®) and its liposomal formulation (AmBisome®). Recent advances in anti-leishmanial chemotherapy include the approval of the first oral drug miltefosine (Impavido®) and the re-discovery of paromomycin (aminosidine).^2^ Paromomycin is the latest drug to be registered for use in India against VL. Limitations of current drugs include significant toxicity, high cost and long treatment courses. Drug resistance to some anti-leishmanial drugs has developed in certain parts of the world with one important focus in the state of Bihar in India.^3^

The advent of new drugs has led to a renewed interest in anti-leishmanial combination chemotherapy as a real possibility.^4^ This strategy is practiced in the treatment of TB, HIV infections and malaria. Potential advantages of a combination chemotherapy approach against VL include decreased toxicity as a result of lower drug doses and/or shorter treatment courses, better patient compliance, lower cost and possibly a reduced likelihood of resistance development.^4,5^

One of the issues in developing drugs against leishmaniasis is delivery of compounds to the parasite which is found within a parasitophorous vacuole (PV) within macrophages. A possible solution is to use polymer–drug conjugates to deliver compounds to the parasite. Polymer conjugates for lysosomotropic delivery are taken into cells by endocytosis and then trafficked through endosomes to lysosomes.^6^ The PV has many similarities to late endosomes/lysosomes and multiple vacuole trafficking pathways can intersect with *Leishmania* PV;^7^ therefore it is likely that the polymer–drug conjugates can also be trafficked to this compartment. Polymer–drug conjugates have been extensively investigated as potential therapeutics (nanomedicines) in the anti-cancer field.^8^ There are a number of different polymers available including synthetic and natural polymers and biodegradable and non-biodegradable polymers. *N*-(2-Hydroxypropyl)methacrylamide (HPMA) copolymer has previously shown promise in the delivery of an anti-leishmanial 8-aminoquinoline.^9,10^ We have recently reported the anti-leishmanial activity of poly(HPMA)–amphotericin B conjugates both in vitro and in vivo.^11^

In a new development, Vicent et al. have reported the design of polymer–drug conjugates for combination therapy in cancer.^12^ Therefore, we decided to investigate combinations of poly(HPMA)–AmB with another drug attached to the polymer that has anti-leishmanial activity. Requirements for attachment to the polymer include a compound with an amine for linking to the polymer. We decided to investigate bisphosphonates. Bisphosphonates have been shown to have anti-leishmanial activity.^13^ Furthermore, some bisphosphonates are registered for the treatment of osteoporosis, indicating their potential pharmaceutical acceptability. We selected alendronate, as this has a suitable amine function for attachment to the polymer. Alendronate is a potential candidate for delivery as a polymer conjugate as it has poor oral bioavailability,^14^ and poor membrane permeability, both of which could potentially be overcome by this strategy. Here, we report on the synthesis and biological evaluation of the first examples of combinatorial poly(HPMA)–drug conjugates as potential therapeutics for VL.

## 2 Results

### 2.1 Synthesis, characterisation and purification of poly(HPMA)–GFLG–AleA and poly(HPMA)–GFLG–AleA–AmB

**Figure 1:**
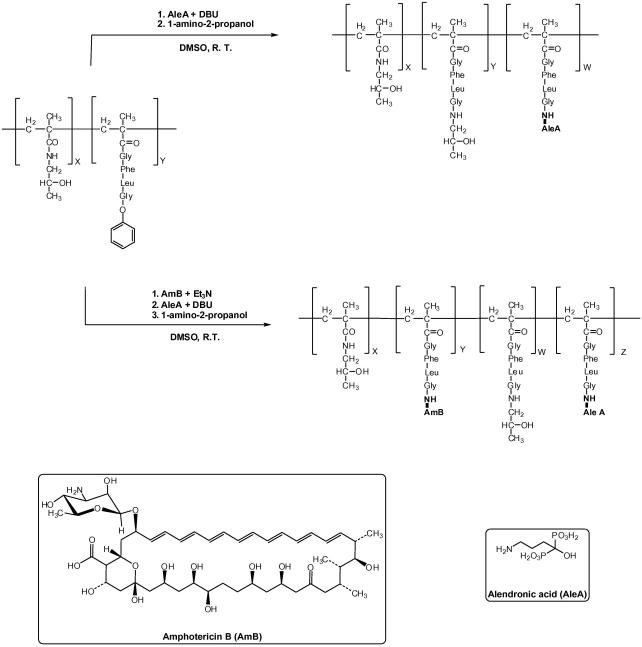
Schematic representation of the synthesis of poly(HPMA)–GFLG–AleA and poly(HPMA)–GFLG–AmB–AleA. The copolymer conjugates were prepared by amidation reaction between the amino group of AleA and/or AmB with the *p*-nitrophenol ester group of the tetrapeptide GFLG side chain of copolymer precursor **1**, and by a final quenching with 1-amino-2-propanol.

A library of poly(HPMA)–GFLG copolymer conjugates was prepared containing alendronic acid (AleA) with or without amphotericin B (AmB) (Table 1). The starting point was commercially available HPMA copolymer containing 9.03 mol % of GlyPheLeuGly (GFLG) side chains, ending with *para*-nitrophenol via ester bond. GlyPheLeuGly was chosen as a linker from the polymer to the drug, as it is known to be cleaved by cathepsin B that is found in the PV; hence compounds should be released in the PV. Copolymer conjugates containing AleA were prepared by displacement of the *para-*nitrophenol by the amino function of AleA in the presence of DBU (Fig. 1). Normally we use DMSO as solvent and triethylamine as base for this coupling. However in this case, the alendronic acid was insoluble in DMSO/triethylamine, but soluble in DMSO/DBU. Unreacted GFLG-*p*-ONp groups were then quenched using 1-amino-2-propanol (AP), to prevent cross-coupling, further reactions and to give a well-defined product. Copolymer conjugates containing AleA and AmB were synthesised in three steps. Firstly, AmB was introduced by reaction of the GFLG-*p*-ONp derivative polymer with the amino group of the drug. Next, the AleA was introduced. Finally, unreacted GFLG-*p*-ONP groups were removed by reaction with 1-amino-2-propanol (Fig. 1). Qualitative and quantitative determination of alendronic acid were performed, respectively, by ^31^P NMR and elemental analysis. The chemical shift in the ^31^P NMR was typical for alendronate.^15^

### 2.2 In vitro efficacy of poly(HPMA)–GFLG–AmB–AleA and corresponding single-drug conjugates against intracellular *Leishmania donovani* in peritoneal mouse macrophages (PEM)

Poly(HPMA)–GFLG–AmB–AleA conjugates were tested for their anti-leishmanial activity against the intracellular amastigote stage in macrophages. Experiments aimed to compare their activity to corresponding single-drug conjugates. For this purpose poly(HPMA)–GFLG–AmB and combinatorial drug conjugates carrying similar weight percentages of amphotericin B were compared. Unbound amphotericin B was included for comparison and Fungizone® used to validate the experiment. Copolymers without drugs attached were also evaluated for control purposes.

**Figure 2:**
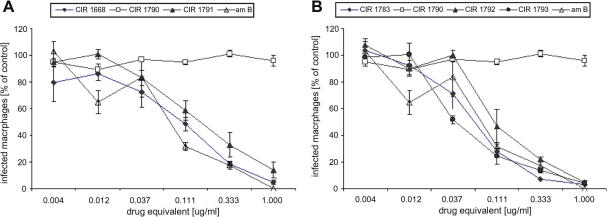
Exposure–response curves for poly(HPMA)–GFLG–AmB, poly(HPMA)–GFLG–AleA and poly(HPMA)–GLFG–AmB–AleA conjugates. (A) compares CIR1668 (9.6 wt % AmB) and CIR1791 (8.2 wt % AmB, 5.5 wt % AleA). (B) compares CIR1783 (16.9 wt % AmB), CIR1792 (13.4 wt % AmB, 3.9 wt % AleA) and CIR1793 (19.6 wt % AmB, 1.8 wt % AleA). CIR1790 contains 7.9 wt % AleA. AmB refers to the free drug tested (77% infected macrophages in control). Corresponding alendronic acid (AleA) equivalents at 1 μg/ml amphotericin B equivalent are as follows: 0.675 μg/ml for CIR1791, 0.290 μg/ml for CIR1792 and 0.092 μg/ml for CIR1793. CIR1790 was used at a starting concentration of 0.675 μg/ml, corresponding to the highest concentration in the combinatorial conjugates (92% infected macrophages in control). Data points give the arithmetic mean ± SEM (*n* = 4) for one representative experiment. Drug equivalent for combination polymers refers to amphotericin B.

In agreement with our previous report^11^ the poly(HPMA)–GFLG–AmB conjugates gave high anti-leishmanial activity, with EC_50_ values below 1 μg/ml and of the same order of magnitude as free amphotericin B and Fungizone® (Table 2, Fig. 2). Thus CIR1668 (9.6% amphotericin B) and CIR1783 (16.9% amphotericin B) showed potent activity. However on its own, poly(HPMA)–GFLG–AleA (CIR1790, 7.9% alendronic acid) showed no significant anti-leishmanial activity. Comparison of CIR1791 (5.5% AleA and 8.2% AmB) with CIR1668 (0% AleA and 9.6% AmB) showed the effect of adding alendronic acid to approximately 8–9% amphotericin B loading; essentially the copolymers were equally active. A similar effect can be seen by comparing CIR1792 (3.9% AleA and 13.4% AmB) and CIR1793 (1.8% AleA and 19.6% AmB) with CIR1783 (0% AleA and 16.9% AmB). These copolymers have relatively high levels of amphotericin B (13–17%) and varying levels of alendronic acid, but showed similar levels of anti-leishmanial activity. Finally comparing CIR1790 (7.9% AleA and 0% AmB) with CIR1791 (5.5% AleA and 8.2% AmB) showed the additive effect of amphotericin B to a loading of 5.5–7.9% alendronic acid; the copolymers went from essentially inactive to a very good level of anti-leishmanial activity.

Hence, no advantage of the combinatorial poly(HPMA)–GFLG–AmB–AleA conjugates was observed as compared to single-drug poly(HPMA)–GFLG–AmB conjugates. Furthermore, there was no significant activity for the poly(HPMA)–GFLG–AleA conjugate within the concentration range tested (Fig. 2).

### 2.3 In vivo efficacy of poly(HPMA)–GFLG–AmB–AleA and poly(HPMA)–GFLG–AmB conjugates against *L. donovani* in the BALB/c mouse model

The in vivo activity of poly(HPMA)–GFLG–AmB–AleA conjugates was evaluated in the BALB/c mouse model at a single dose level of conjugate at 1 mg/kg amphotericin B equivalent × 3 and compared to single-drug conjugates that had shown in vitro activity. The experiment was validated with AmBisome®.

Again we observed high anti-leishmanial activity of both single-drug and combinatorial conjugates, but no advantage of adding alendronic acid was seen (Table 3). In vivo activity of drug conjugates with similar amphotericin B loadings was in the same range for single-drug and combinatorial drug conjugates. CIR1668 (9.6 wt % amphotericin B) and CIR1791 (8.2 wt % amphotericin B, 5.5 wt % alendronic acid) inhibited hepatic parasite burden by 36% and 44%. CIR1783 (16.9 wt % amphotericin B) was the most active conjugate with an inhibition of 77.5%. In comparison CIR1792 (13.4 wt % amphotericin B, 3.9 wt % AleA) and CIR 1793 (19.6 wt % amphotericin B, 1.8 wt % AleA) displayed inhibitions of 54% and 68%. AmBisome® remained the most active formulation and gave an inhibition of 99.5% of hepatic burden. No overt signs of toxicity were recorded in any of the treatment groups and the percentage weight change in treated groups was <1%.

## 3 Discussion

Combination chemotherapy is becoming an attractive strategy for tackling leishmaniasis. In earlier studies combinations of sodium stibogluconate with paromomycin were found to be safe and effective in early trials conducted in India and East Africa.^16–18^ A combination regime of sodium stibogluconate and paromomycin has been employed in Sudan by MSF.^19^ A recent trial of 2-drug chemotherapy, single dose AmBisome® followed by a short course of miltefosine, has shown promising results for this sequential tandem approach against Indian VL.^4^ Additionally various short course multidrug regimes are expected to form part of the visceral leishmaniasis elimination programme.^20^

A single formulation of a fixed dose of two drugs has been described as part of an ideal profile for anti-malarial drug combinations^21^ and recently a hybrid compound derived from mefloquine and artesunate has been reported to be more active than a combination of both single drugs against the malaria parasite.^22^ However, in the case of leishmaniasis, the routes of administration are different for most of the currently approved drugs (po for miltefosine, iv for Fungizone® and AmBisome®, im for sodium stibogluconate and im for paromomycin^2^) and pentavalent antimony has ceased to be a treatment option in India in the state of Bihar due to high resistance levels.^23^ This means fixed-dose combination regimes with ease of administration and using a single route of administration, are problematic in most Leishmania endemic regions, including the region carrying most of the VL burden. Therefore, a delivery system that would guarantee concurrent administration of two or more drugs coupled with the advantage of timely equal delivery at the site of infection, notably *Leishmania* containing macrophages and at a subcellular level the parasitophorous vacuole, does appear attractive.

In this paper, we describe the use of water-soluble polymer–2-drug conjugates for simultaneous delivery of multiple agents. This approach has been successfully undertaken experimentally in the anti-cancer field,^12^ where Vicent et al. simultaneously delivered a cytotoxic agent (doxorubicin) and endocrine therapy (aminoglutethimide). In their study, the drugs showed a synergistic effect when combined on the same polymer, which was not evident when mixtures of polymer conjugates with each agent individually on the polymer backbone were used. Interestingly, the combination appeared to have different release kinetics compared to the individual agents when coupled separately to polymer.

We attached amphotericin B and alendronic acid to the HPMA backbone, obtaining soluble conjugates. The polyene antibiotic amphotericin B complexes with 24-substituted sterols, such as ergosterol in cell membranes, causing channel formation which alters ion balance and results in cell death.^24^ Alendronic acid is a nitrogen-containing bisphosphonate (NBp). NBps have been demonstrated to inhibit isoprenoid biosynthesis at the farnesyl pyrophosphate synthase (FPPS) level of the mevalonate pathway.^25^ NBps, notably alendronate, pamidronate and risedronate, have shown in vitro activity of varying order of magnitude against intracellular *L. donovani* amastigotes and other parasitic protozoa.^13^ In this case, alendronate was reported to have an EC_50_ of 83 μM against intracellular amastigotes. In vivo activity has been demonstrated for pamidronate against experimental CL caused by *L. amazonensis*,^26^ as well as for pamidronate and risedronate against *L. donovani* in the BALB/c mouse model.^27^ NBps are charged molecules with poor oral bioavailability,^14,28^ and once in the body, bisphosphonates are taken up by the skeleton.^29^ Attachment of these compounds to a polymer backbone might result in more favourable cell targeting for *Leishmania*, although a report has indicated that polymer bound alendronate may still be targeted to the bone.^30^ Unfortunately, no activity was seen with the poly(HPMA)–GFLG–AleA copolymer in vitro. It may be that the alendronic acid is not released from the polymer, or that it is not delivered to infected macrophages in sufficient concentration to have an effect.

Although we were able to confirm our previous report of high anti-leishmanial in vitro and in vivo activity of poly(HPMA)–GFLG–AmB conjugates, combinatorial poly(HPMA)–GFLG–AmB–AleA conjugates did not show advantage over the single drug poly(HPMA)–GFLG–AmB polymer conjugates. The failure to observe any increase in drug activity by administration of the two pendant drugs could be due to the nature of alendronic acid and its low potency against *Leishmania*. Indeed free bisphosphonates do not show high intrinsic anti-leishmanial activity apart from risedronate. However the use of risedronate was not possible in this study due to chemistry constraints. Alendronic acid appeared to have a low therapeutic index in vitro with variability between experiments (personal observation) and the alendronic acid containing conjugate without amphotericin B did not show inherent anti-leishmanial activity. Low potency of pendant drugs is an acknowledged factor in the failure of drug delivery systems.^8^ Additionally we do not know at present if drug release from the polymer backbone is necessary for activity, neither how and if amphotericin B and alendronic acid are released. Further experiments would reveal if the drugs are released in the environment of the parasitophorous vacuole. Incubation with cathepsin B^6,31^ or tritosomes to look for drug release^32^ have been reported for investigating drug release from polymer–drug conjugates.

It still has to be noted that single-drug and combinatorial drug conjugates retained high anti-leishmanial activity and the present alendronic acid loading did not appear to have a negative effect on anti-leishmanial drug activity. No apparent signs of toxicity were recorded for either single drug or combinatorial drug conjugates.

## 4 Conclusions

As far as we are aware, this is the first report on anti-leishmanial drug combinations based on a macromolecular drug delivery system for anti-leishmanial chemotherapy. Further investigations into copolymers carrying two pendant drugs as a novel approach for anti-leishmanial combination chemotherapy will be useful. By choosing drugs with a higher inherent anti-leishmanial activity an effective combinatorial drug delivery system might be obtained. The high efficacy of poly(HPMA)–GFLG–AmB conjugates was confirmed.

## 5 Experimental

### 5.1 Materials, instruments and methods

HPMA copolymer with a weight average molecular weight (Mw) of 37,427 g/mol and a polydispersity (Mw/Mw) of 1.58, containing 9.03 mol % GlyPheLeuGly linker activated as the *para-*nitrophenol ester (ONp) (poly(HPMA)–GFLG–ONp) was purchased from Polymer Laboratories Ltd, Church Stretton, UK. Triethylamine was distilled from potassium hydroxide and stored under nitrogen. Amphotericin B with a content of amphotericin A less than 5% was purchased from A.G. Scientific, Inc. San Diego CA. Alendronic acid was purchased from AK Scientific, Inc. Mountain View CA. Other reagents and solvents were purchased from Aldrich and Fluka. All the reactions were carried out at room temperature under argon atmosphere unless specified.

Normal phase thin layer chromatography (TLC) was performed using pre-coated sheets of Silica 60F_254_, using methanol as mobile phase (*R*_f_ of amphotericin B = 0.31; *R*_f_ of 4-nitrophenol and of the polymer = 0.9). Sephadex™ LH20 and dialysis membrane (Molecular Weight Cut Off of 2000, regenerated cellulose tubing Spectra/Por® 6) were purchased from Fisher Scientific.

^1^H NMR spectra were recorded on a Bruker Avance DPX 500 spectrometer. All the signals are described as broad (br). UV–vis spectra were recorded on a Beckman DU 640 spectrophotometer. Solid phase extraction (SPE) high performance liquid chromatography (HPLC) analyses were performed using a Dionex Ultimate 3000 HPLC instrument, using a μBondapack C_18_ 10 μm 125 Å, 300 mm × 4.6 mm I.D. column (Waters). SPE was carried out using Bond Elut® C18 cartridges of 3 ml capacity containing 100 mg of stationary phase (Varian). Microwave experiments were performed in a Biotage Initiator Reactor (Biotage, UK). Elemental analyses were performed by MEDAC Ltd (UK).

### 5.2 Synthesis of polymer–drug conjugates

Syntheses of poly(HPMA)–GFLG–AmB (CIR1668) has been reported recently and that of HMPA–GFLG–AmB (CIR1783) was carried out using our previously reported methodology.^11^

### 5.3 Synthesis of poly(HPMA)–GFLG–alendronic acid conjugate (CIR1790)

To a stirred solution of poly(HPMA)–GFLG–ONp (9.03 mol %) (424.2 mg, 0.22 mmol ONp) in dry DMSO (10.0 ml) was added 896 μl (0.031 mmol) of a solution of alendronic acid (107.0 mg, 0.43 mmol, dissolved in 12.0 ml of DMSO and 323.0 μl of DBU, 2.16 mmol, heated in the microwave reactor at 200 °C for 10 s). The resulting mixture was stirred overnight, quenched with 90 μl of 1-amino-2-propanol (1.17 mmol), diluted with water, afterwards it was dialysed and freeze dried. White foam, 310 mg. Yield based on polymer weight, 73.1%. ^1^H NMR (500 MHz, CD_3_OD); *δ* 1.02–4.53 [br, H of HPMA, glycine, leucine, phenylalanine and 1-amino-2-propanol, and alendronic acid]; *δ* 7.15 and 7.4 [br, Ph, of phenylalanine]. ^31^P NMR (121 MHz, CD_3_OD); *δ* 19.8 [P of PO_3_H_2_ in alendronate)].

### 5.4 Synthesis of poly(HPMA)–GFLG–alendronic acid–AmB conjugates (example described for CIR 1792)

A solution of amphotericin B (72.79 mg dissolved in 1.7 ml of dry DMSO, 0.076 mmol) and triethylamine (11.0 μl, 0.078 mmol) were added to a stirred solution of poly(HPMA)–GFLG–ONp (9.03 mol % ONp) (302.45 mg, 0.15 mmol ONp) in dry DMSO (3.0 ml) with monitoring by normal phase TLC. After two hours more triethylamine was added (11.0 μl, 0.078 mmol). The obtained solution was stirred for one hour more and a solution of alendronic acid was added to it (20.07 mg, 0.078 mmol, dissolved in 2.0 ml of DMSO and 60 μl of DBU, 0.39 mmol, heated in the microwave reactor at 200 °C for 10 s). The resulting solution was stirred for 16 h and quenched with 1-amino-2-propanol (60 μl, 0.78 mmol). The obtained reaction mixture was poured in diethyl ether, the suspension was centrifuged and the supernatant was removed. The pellet (a brown *rubbery* polymer) was dissolved in a minimum amount of methanol and purified by gel filtration using LH20 as stationary phase and methanol as eluent. All the fractions were monitored by UV–vis at 407 nm. The fractions related to the first peak (polymer conjugate) were collected, the solvent was removed and the yellow residue was dissolved in water and freeze dried. The product was a yellow foam 210.0 mg (yield based on polymer weight, 69.5%); ^1^H NMR (500 MHz, CD_3_OD); *δ* 1.01–5.92 [br, H of HPMA, glycine, leucine, phenylalanine, amphotericin B and alendronic acid]; *δ* 6.39 [br, H of double bonds in amphotericin B]; *δ* 7.39 and 7.58 [br, H of Ph in phenylalanine]. ^31^P NMR (121 MHz, CD_3_OD); *δ* 19.7 [P of PO_3_H_2_ in alendronate)].

CIR1791 and CIR1793 were made using the same protocol, but with different quantities of reagents.

### 5.5 Analytical methods

The total amphotericin B content in polymer–drug conjugates was determined using UV–vis spectroscopy and the levels of free unbound, residual amphotericin B in polymer–drug conjugates by SPE-HPLC as previously reported.^11^ The total levels of alendronic acid in polymer–drug conjugates was determined by elemental analysis of phosphorus levels.

### 5.6 In vitro anti-leishmanial activity testing

*L. donovani* strain MHOM/ET/67/L82 was maintained in Syrian hamsters (*Mesocricetus auratus*) and amastigotes harvested from the spleen of an infected animal.

Peritoneal exudate macrophages (PEMs) were harvested from CD1 mice (Charles River Ltd, Margate, UK) by lavage with cold RPMI 1640 medium (Sigma) 24 h after injecting 2 ml of 2% soluble starch (Sigma) into the peritoneal cavity. Macrophages were plated in Lab-tek 16-well chamber slides (Nunc, USA) at a density of 5 × 10^4^ cells/well and left to adhere O/N at 37 °C in a 5% CO_2_–95% humidified air mixture. Adherent PEMs were infected with *L. donovani* amastigotes at a ratio of five amastigotes to one macrophage. After O/N incubation at 37 °C in 5% CO_2_ in humidified air non-phagocytosed amastigotes were removed and 200 μl of threefold serially diluted drug and polymer solutions added to the respective wells. Stock solutions of free amphotericin B, poly(HPMA)–GFLG–AmB conjugates and poly(HPMA)–GFLG–AmB–AleA conjugates were prepared in 100% DMSO (dimethyl sulfoxide, Sigma) at 1 mg/ml amphotericin B equivalent. Fungizone® was reconstituted according to manufacturer’s protocol to obtain a 5 mg/ml stock of amphotericin B. All subsequent dilutions were prepared in RPMI 1640 medium plus 10% hi-FCS. Six concentrations were tested, ranging from 1 to 0.004 μg/ml amphotericin B equivalent. Each drug concentration was tested in quadruplicate. Maximum DMSO concentration in the assays was 0.1%, which had no effect on macrophage parasite clearance.

Infected cultures were incubated for 72 h at 37 °C in a 5% CO_2_–95% humidified air mixture. At experimental endpoint slides were fixed with 100% methanol and stained with 10% Giemsa in water. Percentage inhibitions caused by drug and conjugate activity were determined from the percentage of infected macrophages in relation to a non-treated control upon microscopically counting of 100 macrophages per well. Data were fitted using the non-linear sigmoidal curve-fitting Levenburg Marquardt algorithm and EC_50_/EC_90_ values estimated using Microsoft *xl*fit (ID Business Solution, Guildford, UK). Two independent experiments were performed.

### 5.7 In vivo anti-leishmanial activity testing

All animal experiments were conducted under licence in accordance with UK Home Office regulations. Female Balb/c mice were infected by injection of 2 × 10^7^ amastigotes in 0.2 ml RPMI medium without serum into the tail vein and randomly sorted into groups of five. Solutions of poly(HPMA)–drug conjugates were prepared in PBS (phosphate buffered saline, Sigma). AmBisome® (Gilead Scienes) was reconstituted according to manufacturer’s protocol to yield a stock solution of 4 mg/ml amphotericin B. Subsequent dilutions of AmBisome® were prepared in 5% dextrose. Groups of five mice were dosed intravenously (iv) at an amphotericin B equivalent of 1 mg/kg on days 7, 9 and 11 post-infection with a bolus injection of 0.2 ml using 25 gauge needles. AmBisome® was included at the same dose of 1 mg/kg amphotericin B equivalent. On day 14 post-infection mice were killed and impression smears of livers taken after weighing the organs. Group weights were recorded before and after treatment. Smears were fixed in 100% methanol, stained in 10% Giemsa in water and the number of amastigotes per 500 host cell nuclei counted. The number of Leishman–Donovan units (LDU) was calculated using the formula: LDU = number of parasites per host cell nucleus × organ weight in mg.^33^ Deviations from the number of host cell nuclei given above were taken into account and corrected for by changing the reference base.

Reduction in parasite burden achieved in a particular animal was calculated relative to the mean LDU (*n* = 5) of the control group and expressed as percentage inhibition.

## Figures and Tables

**Table 1:** Polymers prepared and evaluated

		Conjugate	Total AleA (% w/w)	Total AmB (% w/w)	Free AmB (% total drug)
1		Poly(HPMA)–GFLG–ONp		—	—
3	CIR1790	Poly(HPMA)–GFLG–AleA	7.9		
4	CIR1791	Poly(HPMA)–GFLG–AmB–AleA	5.5	8.2	<1.0
5	CIR1792	Poly(HPMA)–GFLG–AmB–AleA	3.9	13.4	<1.0
6	CIR1793	Poly(HPMA)–GFLG–AmB–AleA	1.8	19.6	<1.0
7	CIR1668	Poly(HPMA)–GFLG–AmB	—	9.6	<0.1
8	CIR1783	Poly(HPMA)–GFLG–AmB	—	16.9	<0.1
9	CIR1465	Poly(HPMA)–GFLG–COOH	—	—	—
10	CIR1466	Poly(HPMA)–GFLG–AP	—	—	—

CIR1465, CIR1466 and CIR1668 have been reported previously.^11^

**Table 2:** EC_50_ and EC_90_ values of single and combinatorial poly(HPMA)–GFLG-conjugates against *L. donovani* amastigotes in peritoneal exudate macrophages, for one representative experiment

Conjugate	AmB (wt %)	Al. acid (wt %)	EC_50_ (μg/ml)	EC_90_ (μg/ml)
Amphotericin B	>95	N/A	0.06 (0.04–0.09)	0.44 (0.20–0.68)
Fungizone®	N/A	N/A	0.07 (0.04–0.10)	0.13 (0.11–0.15)
CIR1668	9.63	0	0.09 (0.08–0.10)	0.68 (0.30–1.06)
CIR1790	0	7.88	n.o.	n.o.
CIR1791	8.15	5.49	0.18 (0.09–0.27)	1.18 (0.60–1.76)
CIR1792	13.43	3.92	0.13 (0.09–0.17)	0.36 (0.20–0.52)
CIR1793	19.55	1.83	0.05 (0.03–0.07)	0.22 (0.13–0.31)
CIR1783	16.93	0	0.06 (0.04–0.08)	0.21 (0.14–0.28)
CIR1465	0	0	n.o.	n.o.
CIR1466	0	0	n.o.	n.o.

The infection level in untreated controls was 77%. Values are given as amphotericin B equivalent in μg/ml with 95% confidence intervals in brackets (*n* = 4). Drug loading is given in weight% (wt %). N/A not applicable, n.o. not obtained as CIR1465,^11^ CIR1466^11^ and CIR1790 were inactive within the concentration range tested. No toxicity to the macrophages was observed at the concentrations of copolymer used.

**Table 3:** Inhibition of hepatic parasite burden after treatment with CIR1668, CIR1783, CIR1791, CIR1792 and CIR1793

Code name	Conjugate	AmB loading	Ale acid loading	% Inhibition ± SEM	LDU ± SEM
CIR1668	Poly(HPMA)–GFLG–AmB	9.6 wt %	0	36.1 ± 10.4	1672 ± 139
CIR1783	Poly(HPMA)–GFLG–AmB	16.9 wt %	0	77.5 ± 3.0	1672 ± 139
CIR1791	Poly(HPMA)–GFLG–AmB–Ale acid	8.2 wt %	5.5 wt %	44.4 ± 2.6	1672 ± 139
CIR1792	Poly(HPMA)–GFLG–AmB–Ale acid	13.4 wt %	3.9 wt %	53.8 ± 5.2	1672 ± 139
CIR1793	Poly(HPMA)–GFLG–AmB–Ale acid	19.6 wt %	1.8 wt %	67.9 ± 6.9	1672 ± 139
AmBisome®	n/a	n/a	n/a	99.5 ± 0.2	1672 ± 139

Values presented give the arithmetic mean ± SEM of treated groups (*n* = 5). LDU Leishman–Donovan unit at the experimental endpoint.
